# A Comparative Study on X-ray Shielding and Mechanical Properties of Natural Rubber Latex Nanocomposites Containing Bi_2_O_3_ or BaSO_4_: Experimental and Numerical Determination

**DOI:** 10.3390/polym14173654

**Published:** 2022-09-02

**Authors:** Arkarapol Thumwong, Manchusa Chinnawet, Preawpraw Intarasena, Chanis Rattanapongs, Shinji Tokonami, Tetsuo Ishikawa, Kiadtisak Saenboonruang

**Affiliations:** 1 Department of Materials Science, Faculty of Science, Kasetsart University, Bangkok 10900, Thailand; 2 Department of Applied Radiation and Isotopes, Faculty of Science, Kasetsart University, Bangkok 10900, Thailand; 3 Institute of Radiation Emergency Medicine, Hirosaki University, Aomori 0368564, Japan; 4 Department of Radiation Physics and Chemistry, Fukushima Medical University, Hikarigaoka 9601295, Japan; 5 Kasetsart University Research and Development Institute (KURDI), Kasetsart University, Bangkok 10900, Thailand; 6 Specialized Center of Rubber and Polymer Materials in Agriculture and Industry (RPM), Faculty of Science, Kasetsart University, Bangkok 10900, Thailand; 7 Special Research Unit of Radiation Technology for Advanced Materials (RTAM), Faculty of Science, Kasetsart University, Bangkok 10900, Thailand

**Keywords:** natural rubber latex, Bi_2_O_3_, BaSO_4_, X-ray shielding, gloves, mechanical properties

## Abstract

This work experimentally determined the X-ray shielding and morphological, density, and tensile properties of sulfur-vulcanized natural rubber latex (SVNRL) nanocomposites containing varying content of nano-Bi_2_O_3_ or nano-BaSO_4_ from 0 to 200 phr in 100 phr increments, with modified procedures in sample preparation to overcome the insufficient strength of the samples found in other reports. The experimental X-ray shielding results, which were numerically verified using a web-based software package (XCOM), indicated that the overall X-ray attenuation abilities of the SVNRL nanocomposites generally increased with increasing filler content, with the 0.25-mm-thick SVNRL films containing 200 phr of the filler providing the highest overall X-ray shielding properties, as evidenced by the highest values of lead equivalence (Pb_eq_) of 0.0371 mmPb and 0.0326 mmPb in Bi_2_O_3_/SVNRL nanocomposites, and 0.0326 mmPb and 0.0257 mmPb in BaSO_4_/SVNRL nanocomposites, for 60 kV and 100 kV X-rays, respectively. The results also revealed that the addition of either filler increased the tensile modulus at 300% elongation (M300) and density but decreased the tensile strength and the elongation at break of the Bi_2_O_3_/SVNRL and BaSO_4_/SVNRL nanocomposites. In addition, the modified procedures introduced in this work enabled the developed nanocomposites to acquire sufficient mechanical and X-ray shielding properties for potential use as medical X-ray protective gloves, with the recommended content of Bi_2_O_3_ and BaSO_4_ being in the range of 95–140 phr and 105–120 phr, respectively (in accordance with the requirements outlined in ASTM D3578-19 and the value of Pb_eq_ being greater than 0.02 mmPb). Consequently, based on the overall outcomes of this work, the developed Bi_2_O_3_/SVNRL and BaSO_4_/SVNRL nanocomposites show great potential for effective application in medical X-ray protective gloves, while the modified procedures could possibly be adopted for large-scale production.

## 1. Introduction

High-energy electromagnetic (EM) waves, especially X-rays and gamma rays, are currently utilized in various applications, including the quantification of elements, compounds, and radionuclides contained in commercial products, plants, and foods [1,2,3]; medical and industrial imaging [4,5]; cancer diagnostics and therapy [6,7], and quality control for medical and industrial products [8]. Despite their wide utilization and increasing demand, the adverse effects of excessive exposure to various types of radiation could seriously harm both users and the public, for whom the symptoms may vary from skin reddening, nausea, vomiting, diarrhea, hair loss, and cancer to even death, depending on several factors, such as the exposure dose and duration, the receiver’s age and sex, as well as the levels of sensitivity and responses of the exposed organs to radiation [9,10,11]. Consequently, to reduce or prevent the risk of such adverse effects, a radiation safety principle, namely “As Low As Reasonably Achievable”, or ALARA, must be strictly followed in all facilities, involving the management of the radiation exposure working time and distance, as well as the utilization of appropriate and effective radiation-shielding equipment [12].

Several types of products have been developed and used specifically for X-ray shielding, for which the selection of materials for the equipment has depended on factors such as the nature of usage, specific requirements for intended applications, and the available budget to acquire the equipment. Particularly for applications requiring excellence in flexibility, strength, and elongation at break of the products, as well as the ability to accommodate high content of radiation-protective fillers, natural rubber (NR) composites have emerged as one of the most promising candidates to serve such purposes and needs [13,14,15]. In addition to these properties, NR composites offer other favorable properties, such as being natural products with biodegradability, which is consistent with today’s increasing demand for environmentally friendly materials [16,17]. Examples of NR composites used in X-ray protection include those containing Bi_2_O_3_ [18,19], WO_3_ [20], BaSO_4_ [21,22], Pb [23], and PbO [24] as radiation-protective fillers, for which the fillers can considerably enhance the X-ray attenuation ability of a composite with respect to pristine NR. Among the aforementioned fillers, Bi_2_O_3_ and BaSO_4_ are the two most common lead-free fillers that are suitable to be used as X-ray attenuators due to their economical accessibility, the high atomic numbers (Z) of Bi and Ba (Z = 83 and Z = 56, respectively), and the high densities (ρ) of Bi_2_O_3_ and BaSO_4_ (ρ = 8.9 g/cm^3^ and ρ = 4.5 g/cm^3^, respectively), which result in substantially enhanced interaction probabilities between the materials and incident X-rays [25]. Furthermore, Bi_2_O_3_/NR and BaSO_4_/NR composites, exhibiting comparable or even greater X-ray attenuation abilities than those containing Pb and Pb compounds [26], are considered less hazardous for production and use, as evidenced by the higher permissible exposure limits and thresholds for Bi_2_O_3_ and BaSO_4_ than those for Pb [27].

Due to possible exposure to both primary and secondary X-rays by medical personnel working directly with or close to radioactive sources, the use of appropriate X-ray protective gloves is crucial for their safety. Recently, formulations and processes for the production of X-ray protective gloves have been developed based on sulfur-vulcanized and gamma-vulcanized natural rubber latex (SVNRL and GVNRL, respectively) composites containing nano-Bi_2_O_3_ [28]. The results indicated that while both curing systems offered improved X-ray shielding properties to the samples after the addition of nano-Bi_2_O_3_, only the GVNRL composites had sufficient tensile properties to surpass the requirements for medical examination gloves, according to ASTM D3578-19 [28,29], while the SVNRL composites suffered substantial drops in their strength after the addition of high filler content. This unsatisfactory result was due to the nano-Bi_2_O_3_ molecules obstructing the functionality of the main activators and accelerators during the process of vulcanization, which prevented the complete curing of the samples and, hence, reduced the overall strength of the samples. Consequently, despite the beneficial evidence of nanoparticles used for X-ray protection, the inferior mechanical strength of the SVNRL gloves (a common method to prepare latex gloves commercially [30]) has prevented the implementation of such a procedure in actual large-scale production. Hence, a new method or procedure for sample preparation must be developed to make the production of X-ray protective gloves possible using commonly available technology and equipment.

Hence, the current work aimed to develop novel procedures for the production of SVNRL composites containing either nano-Bi_2_O_3_ or nano-BaSO_4_, with the filler content varying from 0 to 100 and 200 phr, for potential use as medical X-ray protective gloves (the maximum filler content was 200 phr, based on our previous work that indicated the recommended filler content of 90–170 phr for GVNRL composites [28]). The properties of the nanocomposites investigated in this work were: X-ray shielding (based on the linear attenuation coefficient (µ), the mass attenuation coefficient (µ_m_), the half value layer (HVL), and the lead equivalence (Pb_eq_)) and morphological, physical (density), and mechanical (tensile modulus at 300% elongation (M300), tensile strength, and elongation at break). Furthermore, to verify the reliability and correctness of the experimental results for X-ray shielding measurements, the obtained results were compared with those numerically computed using a web-based software package (XCOM [31]), to determine the recommended filler content for the attenuation of 60 kV and 100 kV X-rays, and subsequently compared to the requirements outlined in ASTM D3578-19 and the value of Pb_eq_ > 0.02 mmPb for medical X-ray-protective gloves. The outcomes of this work do not only present new data on SVNRL nanocomposites for X-ray attenuation but also offer improved procedures for sample preparation that would be beneficial and suitable for actual production at larger scales.

## 2. Experimental Section

### 2.1. Materials and Chemicals

**Figure 1:**
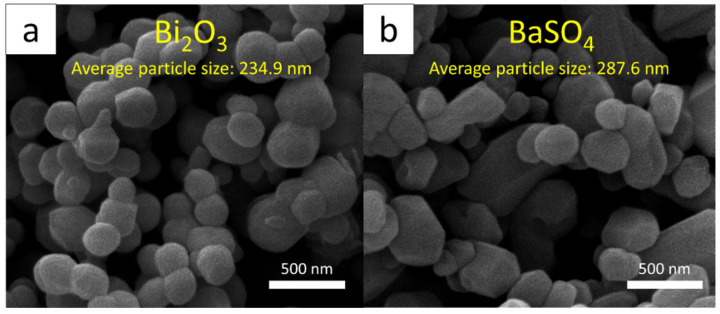
SEM images of (**a**) nano-Bi_2_O_3_ and (**b**) nano-BaSO_4_ (60,000× magnification).

High-ammonia natural rubber latex (HA-NRL) samples, with total solid and dry rubber content of 61.0% (ISO 124: 2014) and 60.3% (ISO 126: 2005), respectively, were supplied by the Office of Rubber Authority of Thailand (RAOT), Bangkok, Thailand. Names, contents, and the roles of chemicals used for the sample preparation are shown in Table 1. Nano-Bi_2_O_3_ and nano-BaSO_4_ were obtained from Shanghai Ruizheng Chemical Technology Co., Ltd. (Shanghai, China), distilled water was supplied by the Faculty of Science, Kasetsart University (Bangkok, Thailand), and other chemicals were supplied by the RAOT (Bangkok, Thailand). The images of nano-Bi_2_O_3_ and nano-BaSO_4_, taken using a scanning electron microscope (SEM; Quanta 450 FEI: JSM-6610LV, Eindhoven, The Netherlands), are shown in Figure 1, indicating that the average particle sizes of nano-Bi_2_O_3_ and nano-BaSO_4_ were 234.9 nm and 287.6 nm, respectively, as determined using the ImageJ software version 1.50i. It should be noted that in order to improve the compatibility between the added chemicals and the NRL matrix, all chemicals used in this work (except KOH and Teric 16A16) were prepared using a stainless-steel ball mill by diluting each pure chemical with vultamol, bentonite, and distilled water for 72 h (final weight content of the chemical: vultamol: bentonite: distilled water was 50:1:1:48). It should be noted that the nanoparticles of Bi_2_O_3_ and BaSO_4_ were selected for this investigation due to their superior radiation-shielding and mechanical properties in the nanocomposites in comparison with those containing microparticles at the same filler content found in previous reports [32,33].

### 2.2. Preparation of SVNRL Mixture

NRL was mechanically stirred using an automatic top stirrer (Eurostar 60 digital, IKA, Bangkok, Thailand) at a rotation speed of 300 rpm for 60 min. Then, all chemicals listed in Table 1 (except nano-Bi_2_O_3_ and nano-BaSO_4_) were consecutively added to the stirred NRL (from top to bottom order), with a 2 min interval between each chemical, and the stirring was continued for another 60 min. Then, the NRL mixture was stored in a closed container at room temperature for 72 h before the addition of the nano-Bi_2_O_3_ or nano-BaSO_4_ to the NRL mixture. The mixture was continuously stirred for another 60 min and kept in a closed container for further use. It should be noted that this step (adding nano-Bi_2_O_3_/nano-BaSO_4_ after the pre-vulcanization process of 72 h) was different from our previous work [28]. This procedure was modified to reduce the effects of nano-Bi_2_O_3_/nano-BaSO_4_ on obstructing the functionality of the main activators and accelerators during vulcanization, which helped the SVNRL mixture to achieve a higher degree of curing that could potentially improve the overall mechanical properties of the nanocomposites [28].

### 2.3. Preparation of Nano-Bi_2_O_3_/SVNRL and Nano-BaSO_4_/SVNRL Gloves

**Figure 2:**
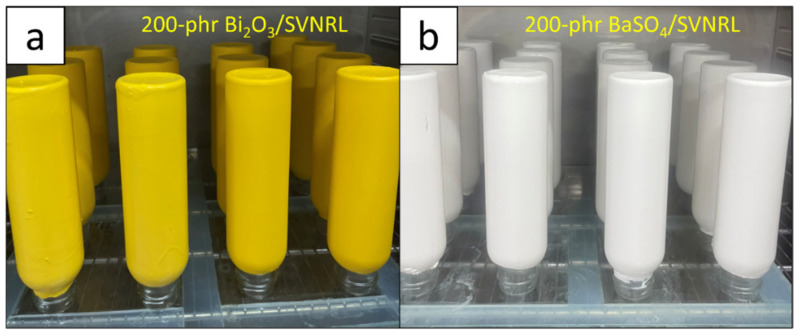
Images showing physical appearance and color of (**a**) nano-Bi_2_O_3_/SVNRL and (**b**) nano-BaSO_4_/SVNRL composites containing 200 phr of their respective fillers.

The procedure to prepare nano-Bi_2_O_3_/SVNRL and nano-BaSO_4_/SVNRL gloves followed the steps outlined in our previous work [28]. In summary, after thorough washing, the ceramic molds were oven-dried at 70 °C for 40 min, dipped in a 35% coagulant consisting of Ca(NO_3_)_2_, Teric 16A16, 50% CaCO_3_, and distilled water (RAOT, Bangkok, Thailand) with the final weight content of 35.0:0.1:5.0:59.9, respectively, for 5 sec, and oven-dried again at 70 °C for 2 min. Then, the dried molds were dipped in the nano-Bi_2_O_3_/SVNRL or nano-BaSO_4_/SVNRL mixture for 40 sec, carefully flicked and rotated (at least 3 times), and oven-dried at 70 °C for 5 min. The molds were dipped in 70 °C distilled water for 5 min to rinse off all remaining chemicals and dried again at 100 °C for 40 min. The nano-Bi_2_O_3_/SVNRL or nano-BaSO_4_/SVNRL gloves were peeled off the molds and processed using chlorination to remove any powder that remained on the surface of the gloves [28]. Figure 2 shows images of the prepared nano-Bi_2_O_3_/SVNRL and nano-BaSO_4_/SVNRL samples containing 200 phr of the fillers, which clearly indicate smooth and uniform surfaces, while the colors of the nano-Bi_2_O_3_/SVNRL and nano-BaSO_4_/SVNRL samples were yellow and white, respectively (the same as the colors of the nano-Bi_2_O_3_ and nano-BaSO_4_ particles).

### 2.4. Characterization

#### 2.4.1. X-ray Shielding Properties

**Figure 3:**
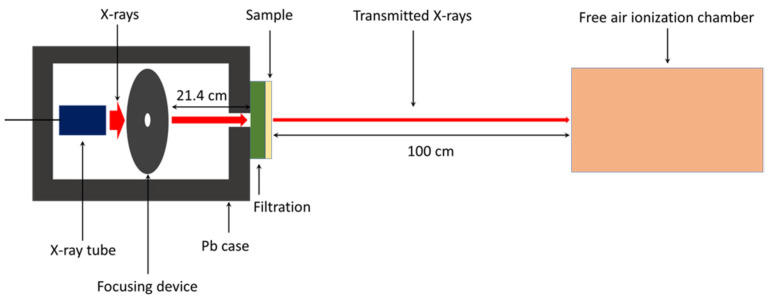
Schematic setup for X-ray shielding measurement.

The X-ray shielding properties of the Bi_2_O_3_/SVNRL and BaSO_4_/SVNRL nanocomposites were investigated at the Secondary Standard Dosimetry Laboratory (SSDL), the Office of Atoms for Peace (OAP), Bangkok, Thailand. The X-ray shielding parameters of interest were the X-ray transmission ratio (I/I_0_), the linear attenuation coefficient (µ), the mass attenuation coefficient (µ_m_), the half value layer (HVL), and the lead equivalence (Pb_eq_), with their relationships shown in Equations (1)–(4):

(1) $$I=I_{0}e^{-\mu x}$$

(2) $$\mu_{m}=\frac{\mu }{\rho }$$

(3) $$\mathrm{HVL}=\frac{\ln\left(2\right)}{\mu }$$

(4) $${\mathrm{Pb}}_{\mathrm{eq}}=\frac{\mu x}{\mu_{\mathrm{Pb}}}$$

where I_0_ is the intensity of incident X-rays, I is the intensity of transmitted X-rays, x is the thickness, µ is the linear attenuation coefficient, µ_m_ is the mass attenuation coefficient, ρ is the density, HVL is the half value layer, Pb_eq_ is the lead equivalence, and µ_Pb_ is the linear attenuation coefficient of a pure lead sheet. For Equation (4), the values of µ_Pb_ were 63.06 cm^−1^ and 25.99 cm^−1^ for the X-ray energies of 45 keV and 80 keV, respectively, numerically determined using the XCOM software (National Institute of Standards and Technology, Gaithersburg, MD, USA). These were the average values of incident X-rays emitted from an X-ray tube with supplied voltages of 60 and 100 kV, respectively, in our setup. The X-rays were collimated using a 1 mm pinhole to achieve a narrow-beam setup and pointed directly to the center of the 0.25-mm-thick SVNRL nanocomposites. The transmitted X-rays were detected and counted using a free air ionization chamber (Korea Research Institute of Standards and Science, KRISS; Daejon, Korea) that was powered by a high-voltage power unit (Keithley 247, Cleveland, OH, USA) and connected to an electrometer (Keithley 6517B, Cleveland, OH, USA). The X-rays used in this work were controlled by an X-ray system (YXLON MGC41, Hudson, NY, USA) and the energies were selected based on ISO 4037-1:2019. The schematic setup for the X-ray shielding measurement is shown in Figure 3 [25].

To verify the correctness and reliability of the experimental results, the numerical determination based on the XCOM software was conducted at X-ray energies of 45 keV and 80 keV and the results were compared with those obtained experimentally [34]. Notably, since XCOM provided only the value of µ_m_, the density (ρ) for each formulation, which was used for the calculation of µ, HVL, and Pb_eq_, was theoretically determined using Equation (5):

(5) $$\rho =\frac{C_{\mathrm{NR}}+C_{F}}{\frac{C_{\mathrm{NR}}}{\rho_{\mathrm{NR}}}+\frac{C_{F}}{\rho_{F}}}$$

where ρ_NR_ (ρ_F_) is the density of NR (radiation-protective filler) and C_NR_ (C_F_) is the content of the NR (radiation-protective filler).

#### 2.4.2. Morphology and Density Measurement

The morphology, the dispersion of nano-Bi_2_O_3_ and BaSO_4_ particles, and the dispersion of Bi and Ba elements in the SVNRL composites were determined using scanning electron microscopy (SEM) with energy-dispersive X-ray spectroscopy (EDX; Quanta 450 FEI: JSM-6610LV, Eindhoven, the Netherlands). All samples were coated with gold using a sputter coater (Quorum SC7620: Mini Sputter Coater/Glow Discharge System, Laughton, UK) prior to the SEM-EDX images being taken.

The density of each sample was determined using a densitometer (MH-300A, Shanghai, China) with a precision of 0.001 g/cm^3^. The determination was carried out based on the Archimedes principle [35].

#### 2.4.3. Mechanical Properties

The tensile properties, consisting of tensile modulus at 300% elongation (M300), tensile strength, and elongation at break, were determined using a universal testing machine (TM Tech, TM-G5K, Bangkok, Thailand) according to ASTM D412-06 standard testing. The tensile testing speed used for all samples was 500 mm/min.

### 2.5. Determination of Recommended Filler Content for Medical X-ray Protective Gloves

The determination of the recommended filler content for the production of medical X-ray protective gloves based on Bi_2_O_3_/SVNRL and BaSO_4_/SVNRL nanocomposites was conducted by comparing the X-ray shielding properties of the 0.25-mm-thick samples, with a minimum required Pb_eq_ value of 0.02 mmPb, as well as their tensile properties, with those outlined in ASTM D3578-19, which states that, for medical examination gloves, the tensile strength and the elongation at break must be higher than 14 MPa and 650%, respectively [29]. Then, ranges of filler content that provided sufficient X-ray shielding and tensile properties in accordance with the above requirements could be selected and recommended for actual use.

## 3. Results and Discussion

### 3.1. Density

The densities of the pristine SVNRL, nano-Bi_2_O_3_/SVNRL, and nano-BaSO_4_/SVNRL composites are shown in Table 2. The results revealed that the densities of the samples increased with increasing filler content, with nano-Bi_2_O_3_/SVNRL having slightly higher densities than nano-BaSO_4_/SVNRL with the same filler content. This was due to the much higher densities of Bi_2_O_3_ and BaSO_4_ compared to the pristine SVNRL (ρ_NR_ = 0.93 g/cm^3^, ρ_Bi___2_O___3__ = 8.9 g/cm^3^, and ρ_BaSO___4__ = 4.5 g/cm^3^), leading to enhanced overall densities of the composites [36]. Notably, these density results were later used for the calculation of µ_m_ from I/I_0_ and µ (Equations (1) and (2)).

### 3.2. X-ray Shielding Properties

Table 3 shows the values of µ, µ_m_, HVL, and Pb_eq_ of the pristine SVNRL, nano-Bi_2_O_3_/SVNRL, and nano-BaSO_4_/SVNRL composites, at the X-ray supplied voltages of 60 kV and 100 kV. The results indicated that the overall X-ray shielding abilities of the SVNRL nanocomposites increased with increasing filler content, as seen by the highest values of µ, µ_m_, and Pb_eq_, and the lowest values of HVL, observed in samples containing 200 phr of the fillers. Furthermore, Table 3 shows that the ability to attenuate X-rays of the nanocomposites was reduced at higher X-ray energies, as evidenced by the lower values of µ, µ_m_, and Pb_eq_ and the higher values of HVL observed in the samples tested using 100 kV X-rays.

The positive relationship between the filler content and X-ray shielding properties was due to the relatively larger Z values of Bi and Ba compared to the C and H in NR, as well as the higher densities of Bi_2_O_3_ and BaSO_4_ compared to NR. These characteristics greatly enhanced the interaction probabilities between incident X-rays and the materials through the dominant and effective photoelectric absorption, which is related to the photoelectric cross-section (σ_pe_) and Z, as shown in Equation (6):

(6) $$\sigma_{\mathrm{pe}}\propto \;\frac{Z^{n}}{{\left(h\nu \right)}^{3}}$$

where h is Planck’s constant and ν is the frequency of the incident X-rays that is directly proportional to the energy, via Equation (7):

(7) E=hν

**Figure 4:**
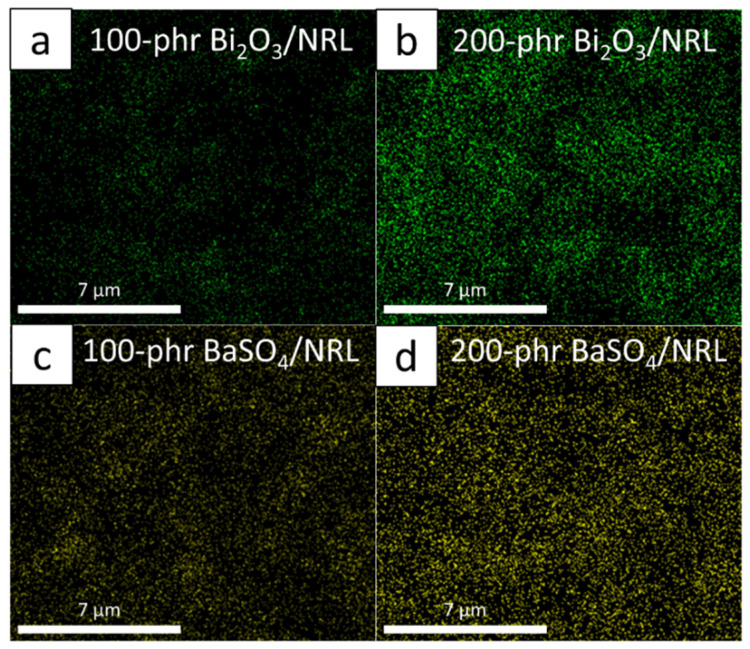
Dispersion of (**a**,**b**) Bi and (**c**,**d**) Ba elements in (**a**) 100 phr Bi_2_O_3_/SVNRL, (**b**) 200 phr Bi_2_O_3_/SVNRL, (**c**) 100 phr BaSO_4_/SVNRL, and (**d**) 200 phr BaSO_4_/SVNRL composites. The images were taken using SEM-EDX with 10,000× magnification.

As depicted in Equation (6), the addition of Bi_2_O_3_ and BaSO_4_ in the SVNRL matrix led to higher numbers of heavy elements (Bi and Ba) available in the composites, resulting in larger σ_pe_ values and, hence, a better ability to attenuate incident X-rays [37]. The increases in the numbers of Bi and Ba elements in the SVNRL composites containing 200 phr of the fillers could be illustrated by considering elemental mapping obtained using the SEM-EDX images (Figure 4), with Figure 4b,d showing the elemental distributions for samples with 200 phr filler and revealing higher concentrations of Bi and Ba atoms, respectively, than in Figure 4a,c, which represent the elemental distributions for samples with 100 phr filler, respectively.

**Figure 5:**
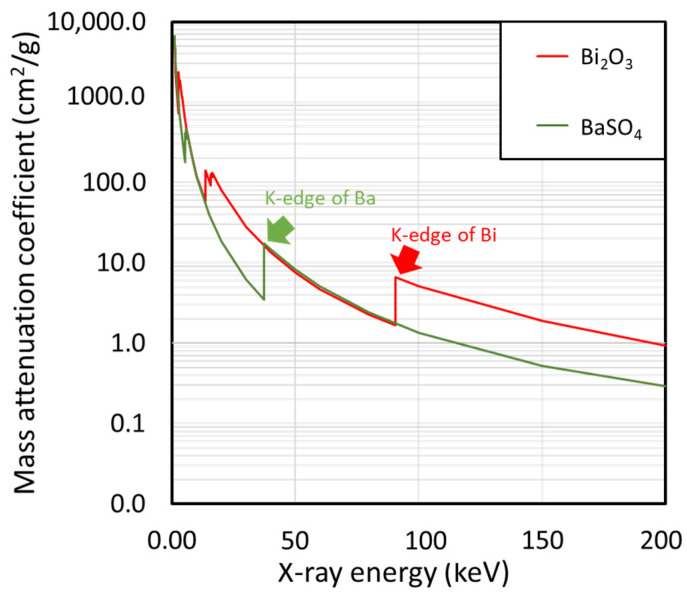
Mass attenuation coefficients (µ_m_) of Bi_2_O_3_ and BaSO_4_ at varying X-ray energies (1–200 keV), determined using XCOM.

Another interesting result shown in Table 3 was that nano-Bi_2_O_3_/SVNRL had slightly higher X-ray shielding properties than the nano-BaSO_4_/SVNRL composites at both supplied voltages. This behavior could be explained by comparing the values of µ_m_ for Bi_2_O_3_ and BaSO_4_, obtained from XCOM at various X-ray energies (Figure 5), which indicated that the µ_m_ values for both Bi_2_O_3_ and BaSO_4_ were similar at the 45 keV X-rays (representing the average X-ray energy of those emitted from the X-ray tube with a supplied voltage of 60 kV), while Bi_2_O_3_ clearly had a higher µ_m_ than that of BaSO_4_ at the 80 keV X-rays (representing the average X-ray energy of those emitted from the X-ray tube with a supplied voltage of 100 kV), leading to the more pronounced enhancement in X-ray attenuation ability in nano-Bi_2_O_3_/SVNRL composites. Notably, although both Bi_2_O_3_ and BaSO_4_ had similar µ_m_ values at the 45 keV X-rays, the densities of nano-Bi_2_O_3_/SVNRL were greater compared to those of nano-BaSO_4_/SVNRL (Table 2), leading to greater amplification of the overall X-ray shielding properties in nano-Bi_2_O_3_/SVNRL (determined at the same filler content). This phenomenon could also be mathematically explained using Equation (2), which implies a direct relationship between µ and ρ. Notably, the sharp increases in µ_m_ at particular X-ray energies in Figure 5 (such as 37.4 keV and 90.5 keV) were due to the K-absorption (K-edge) and L-absorption (L-edge) of Ba and Bi (the X-ray energies that are slightly above the binding energy of the electron shell of the atoms), for which the σ_pe_ or the interaction probabilities between incident X-rays and the compounds abruptly increased at these energies [38].

In addition, Table 3 suggests that the X-ray shielding properties of pristine SVNRL, nano-Bi_2_O_3_/SVNRL, and nano-BaSO_4_/SVNRL composites at the 60 kV X-rays were greater than those at the 100 kV X-rays. This behavior could be explained using Equation (6), which implies that σ_pe_ is inversely proportional to ν^3^ or E^3^, resulting in less interaction probabilities with incident X-rays at higher energies [39]. The dependence of σ_pe_ could also be observed in Figure 5, which reveals overall decreases in the µ_m_ values of Bi_2_O_3_ and BaSO_4_ with increasing X-ray energies.

**Figure 6:**
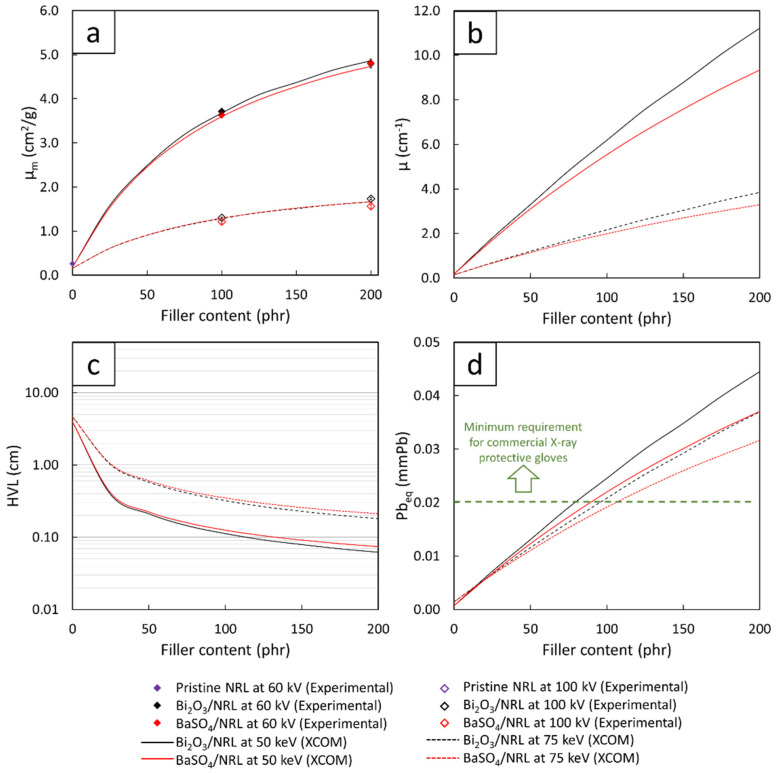
(**a**) Mass attenuation coefficients (µ_m_), (**b**) linear attenuation coefficients (µ), (**c**) half value layer (HVL), and (**d**) lead equivalence (Pb_eq_) of Bi_2_O_3_/SVNRL and BaSO_4_/SVNRL composites containing varying filler content from 0 to 200 phr. The dotted line in (**d**) represents the minimum requirement for commercial X-ray protective gloves.

To verify the correctness and reliability of the experimental results, the µ_m_ values of pristine SVNRL, nano-Bi_2_O_3_/SVNRL, and nano-BaSO_4_/SVNRL composites at filler content of 100 phr and 200 phr were compared with those numerically determined using XCOM (Figure 6a). The comparison indicated strong agreement between the µ_m_ values obtained experimentally and numerically, with the percentage of difference being less than 2% for samples containing 0 and 100 phr of the fillers and being less than 7% for samples containing 200 phr of the fillers. The discrepancies between the two results could have been due to several factors, such as the fact that the experimental X-ray energies emitted from the X-ray tube were actually in spectra, with the average energies being around 45 keV and 80 keV (rather than discrete energies, as in the case of XCOM), which could cause deviations in the X-ray shielding measurements [40,41]. Nonetheless, the small percentages of difference (less than 7%) implied that the experimental results were reliable and the µ_m_ values obtained from XCOM could be further used for the prediction of µ, HVL, and Pb_eq_ values for all filler content values in the range 0–200 phr.

Figure 6b,d, which show the numerical values of µ, HVL, and Pb_eq_, determined using XCOM of the Bi_2_O_3_/SVNRL and BaSO_4_/SVNRL composites with varying filler content from 0 to 200 phr, confirm the dependence of the X-ray shielding properties of the samples on the filler type and content, as well as the X-ray energy, shown in Table 3. For Figure 6d, the results implied that Bi_2_O_3_/SVNRL composites required less filler content to meet the minimum requirement of Pb_eq_ being greater than 0.02 mmPb compared to those from the BaSO_4_/SVNRL composites (determined at the same X-ray energy). Again, these behaviors were observed due to the higher values for µ_m_ (Figure 5) and ρ of Bi_2_O_3_ than those of BaSO_4_, which made the former a better X-ray attenuator than the latter [29,42].

### 3.3. Mechanical Properties

**Figure 7:**
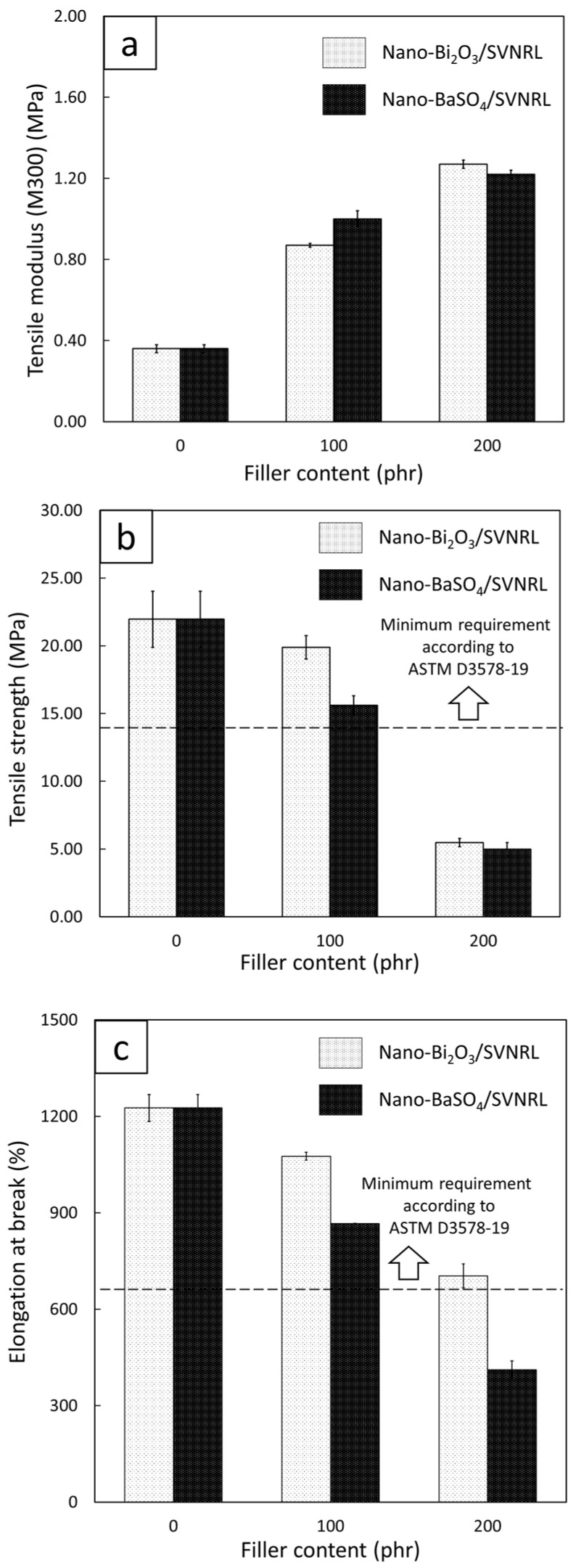
(**a**) Tensile modulus at 300% elongation (M300), (**b**) tensile strength, and (**c**) elongation at break of nano-Bi_2_O_3_/SVNRL and nano-BaSO_4_/SVNRL composites. The dotted lines in (**b**) and (**c**) represent the minimum requirements for tensile strength and elongation at break, respectively, for medical examination gloves according to ASTM D3578-19.

**Figure 8:**
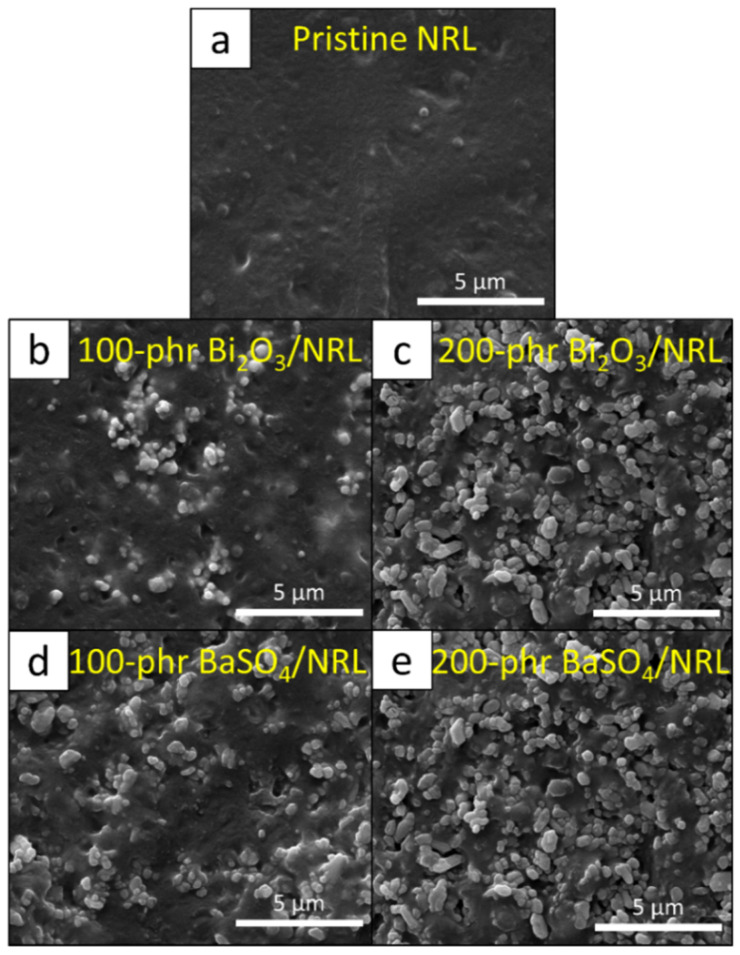
SEM images showing morphology and particle dispersion for (**a**) pristine SVNRL, (**b**) 200 phr Bi_2_O_3_/SVNRL, (**c**) 200 phr Bi_2_O_3_/SVNRL, (**d**) 100 phr BaSO_4_/SVNRL, and (**e**) 200 phr BaSO_4_/SVNRL composites (10,000× magnification).

Figure 7 shows the tensile properties, including tensile modulus at 300% elongation, tensile strength, and elongation at break, of the nano-Bi_2_O_3_/SVNRL and nano-BaSO_4_/SVNRL composites. The results indicated that increases in the filler content led to an increase in the tensile modulus but decreases in the tensile strength and elongation at break. The increase in tensile modulus after the addition of the fillers to SVNRL could have been due to the high rigidity of the nano-Bi_2_O_3_ and nano-BaSO_4_ particles, which enhanced the overall rigidity and, subsequently, the tensile modulus of the nanocomposites [43,44]. On the other hand, the addition of the nano-Bi_2_O_3_ and nano-BaSO_4_ particles resulted in reductions in the tensile strength and elongation at break, probably due to the poor interfacial compatibility between the fillers and the NRL matrix (rubber–filler interactions), which led to visible voids inside the matrix [45]. Another factor that could have contributed to the decreases in the properties was the increase in filler–filler interactions at higher filler content, which resulted in higher particle agglomeration and worse particle dispersion in samples with 200 phr filler content (Figure 8c,e) than in samples with 0 and 100 phr filler content (Figure 8a,b,d) [46].

Figure 7 also reveals that the nano-Bi_2_O_3_/SVNRL composites had higher tensile strength and elongation at break than the nano-BaSO_4_/SVNRL composites, determined at the same filler content. This was mainly due to the higher density of nano-Bi_2_O_3_ particles than nano-BaSO_4_ particles; hence, when both fillers were added to the samples at the same weight content, less volume of nano-Bi_2_O_3_ would be actually added to the composites, resulting in fewer voids and less particle agglomeration created in the nano-Bi_2_O_3_/SVNRL composites. Nonetheless, Figure 7b,c imply that both SVNRL nanocomposites containing less than 100 phr filler had higher experimental values of tensile strength and elongation at break than those outlined in ASTM D3578-19 for medical examination gloves (greater than 14 MPa and 650%, respectively, represented as horizontal dotted lines in this figure). Notably, these mechanical results could be further considered along with the results from the X-ray shielding measurement to determine the recommended filler content that allowed the nanocomposites to satisfy all the requirements for medical X-ray protective gloves.

As mentioned in the experimental section above, the current work modified the procedure for sample preparation by postponing the addition of nano-Bi_2_O_3_ or nano-BaSO_4_ until after the completion of rubber vulcanization (72 h after sulfur was added to the SVNRL mixture). The effects of this improved procedure on the tensile strengths of the samples are shown in Table 4, which indicates that the current tensile strengths of the nano-Bi_2_O_3_/SVNRL composites were higher than those in a previous work for all nano-Bi_2_O_3_ contents investigated [28], especially for the 100 phr content, which showed an almost 3-fold increase in the values. This improvement in tensile strength could have been due to the postponed addition of nano-Bi_2_O_3_ reducing the obstruction effects of the filler on the functionality of the main activators and accelerators, allowing higher degrees of vulcanization to occur prior to the addition of nano-Bi_2_O_3_, which consequently improved the overall strengths of the samples [26]. This outcome would be crucial for the actual production of medical X-ray protective gloves based on SVNRL as the achieved tensile strengths were greater than the strength requirement (ASTM D3578-19), which was unobtainable in the previous report.

### 3.4. Determination of Recommended Filler Content

**Figure 9:**
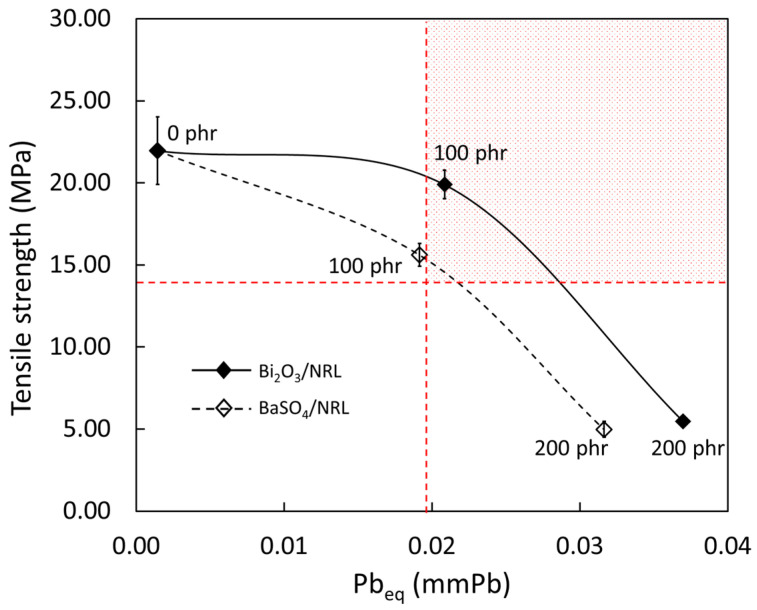
Relationship between tensile strength and Pb_eq_ (from XCOM) of nano-Bi_2_O_3_/SVNRL (solid line) and nano-BaSO_4_/SVNRL (dotted line) composites. The shaded area in the top-right corner indicates the conditions for which samples satisfy both tensile strength and Pb_eq_ requirements for medical X-ray protective gloves (ASTM D3578-19).

To determine the recommended filler content for the actual production of medical X-ray protective gloves based on the requirements outlined in ASTM D3578-19 (tensile strength > 14 MPa) and ensuring a value of Pb_eq_ > 0.02 mmPb, the relationships between the experimental tensile strength and Pb_eq_ of the nano-Bi_2_O_3_/SVNRL and nano-BaSO_4_/SVNRL composites were plotted, as shown in Figure 9, with interpolation between data points. While most of the formulations investigated in this work did not simultaneously satisfy both the X-ray shielding and mechanical requirements, the samples containing approximately 95–140 phr of nano-Bi_2_O_3_ and 105–120 phr of nano-BaSO_4_ offered sufficient characteristics to satisfy the requirements; thus, these filler ranges could be regarded as the recommended filler content levels. Notably, the nano-Bi_2_O_3_/SVNRL composites had larger ranges of recommended filler content than the nano-BaSO_4_/SVNRL composites, which could have been due to the greater levels of X-ray attenuation ability and overall mechanical strength found in the nano-Bi_2_O_3_/SVNRL composites. In addition, these findings confirmed the useability of Bi_2_O_3_ and BaSO_4_ as effective fillers for radiation protection, which were also found in other shielding materials such as glasses and concrete [47,48,49,50].

## 4. Conclusions

This work developed medical X-ray protective gloves based on nano-Bi_2_O_3_/SVNRL and nano-BaSO_4_/SVNRL composites, with varying filler content of 0 to 200 phr in 100 phr increments. The results suggested that the increases in filler content increased the values of µ, µ_m_, HVL, Pb_eq_, density, and tensile modulus at 300% elongation but decreased the tensile strength and elongation at break of the nanocomposites. The experimental results of X-ray shielding measurement were also numerically verified using XCOM, which indicated strong agreement between the two methods (less than 7% difference), implying the reliability and correctness of the results. Furthermore, after considering the X-ray shielding and mechanical properties of both composites, nano-Bi_2_O_3_/SVNRL with filler content of 95–140 phr and nano-BaSO_4_/SVNRL with filler content of 105–120 phr satisfied the minimum requirements of Pb > 0.02 mmPb and tensile strength > 14 MPa outlined in the commercial X-ray protective gloves standard and ASTM D3578-19, respectively. In addition, the modified sample preparation procedures introduced in this work resulted in improved tensile properties of the SVNRL composites (not obtainable in the previous work), potentially making the method suitable for implementation in actual large-scale production.

## Figures and Tables

**Table 1:** Material formulations of SVNRL nanocomposites and their chemical names, content, and roles.

Chemical	Content (phr)	Role
50% *w*/*w* nano-Bi_2_O_3_ or nano-BaSO_4_	0, 100, and 200	X-ray protective filler
10% *w*/*w* potassium hydroxide (KOH)	0.2	Stabilizer
10% *w*/*w* Teric 16A16	0.02	Stabilizer
50% *w*/*w* sulfur (S)	0.8	Crosslinking agent
50% *w*/*w* zinc diethyl dithiocarbamate (ZDEC)	0.4	Accelerator
50% *w*/*w* zinc-2-mercaptobenzthiazole (ZMBT)	0.4	Accelerator
50% *w*/*w* titanium dioxide	1.0	Pigment
50% *w*/*w* wingstay-L	1.0	Antioxidant
50% *w*/*w* zinc oxide (ZnO)	1.0	Activator
Distilled water (H_2_O)	170.5	Solvent

**Table 2:** Densities of pristine SVNRL, nano-Bi_2_O_3_/SVNRL, and nano-BaSO_4_/SVNRL composites containing filler content of 0, 100 phr, and 200 phr.

Sample	Filler Content (phr)	Density (g/cm^3^)
Pristine SVNRL	0	0.93 ± 0.01
Nano-Bi_2_O_3_/SVNRL	100	1.67 ± 0.02
	200	1.95 ± 0.01
Nano-BaSO_4_/SVNRL	100	1.44 ± 0.01
	200	1.71 ± 0.02

**Table 3:** Linear attenuation coefficients (µ), mass attenuation coefficients (µ_m_), half value layer (HVL), and lead equivalence (Pb_eq_) of pristine SVNRL, nano-Bi_2_O_3_/SVNRL, and nano-BaSO_4_/SVNRL composites, at X-ray supplied voltages of 60 kV and 100 kV.

Properties	X-Ray Supplied Voltage	Pristine SVNRL	Bi_2_O_3_/SVNRL		BaSO_4_/SVNRL	
			100 phr	200 phr	100 phr	200 phr
µ (cm^−1^)	60 kV	0.24 ± 0.01	6.19 ± 0.20	9.36 ± 0.38	5.23 ± 0.12	8.23 ± 0.13
	100 kV	0.18 ± 0.01	2.17 ± 0.07	3.38 ± 0.13	1.75 ± 0.01	2.67 ± 0.06
µ_m_ (cm^2^/g)	60 kV	0.26 ± 0.01	3.71 ± 0.12	4.80 ± 0.19	3.63 ± 0.08	4.81 ± 0.07
	100 kV	0.19 ± 0.01	1.30 ± 0.04	1.74 ± 0.07	1.21 ± 0.01	1.56 ± 0.04
HVL (cm)	60 kV	2.87 ± 0.01	0.11 ± 0.01	0.07 ± 0.01	0.13 ± 0.01	0.08 ± 0.01
	100 kV	3.84 ± 0.09	0.32 ± 0.01	0.20 ± 0.01	0.40 ± 0.01	0.26 ± 0.01
Pb_eq_ (mm Pb)	60 kV	0.0010 ± 0.0001	0.0245 ± 0.0008	0.0371 ± 0.0015	0.0207 ± 0.0005	0.0326 ± 0.0005
	100 kV	0.0017 ± 0.0001	0.0209 ± 0.0006	0.0326 ± 0.0012	0.0168 ± 0.0001	0.0257 ± 0.0006

**Table 4:** Mean (± standard deviation) tensile strength of nano-Bi_2_O_3_/SVNRL composites in current work compared to previous work [28].

Bi_2_O_3_ Content (phr)	Tensile Strength (MPa)	
	Current Work	Previous Work [28]
100	19.90 ± 0.87	7.23 ± 1.49
200	6.85 ± 0.75	5.47 ± 0.30
